# Advance in the application of metabolomics technology in poultry

**DOI:** 10.3389/fvets.2024.1501630

**Published:** 2024-12-09

**Authors:** Meimei Zhang, Manhua You, Ning Ma, Jiancun Lv

**Affiliations:** ^1^College of Veterinary Medicine, Huazhong Agricultural University, Wuhan, China; ^2^Veterinary Biological Technology Innovation Center of Hebei Province, College of Veterinary Medicine, Hebei Agricultural University, Baoding, China; ^3^College of Veterinary Medicine, Hebei Agricultural University, Baoding, China

**Keywords:** metabolomics, application, egg, pathway, metabolites, poultry

## Abstract

Metabolomics is a science that takes small molecular metabolites in organisms as the research object and determines the dynamic changes of metabolites at the overall level through a variety of modern analytical techniques. At present, metabolomics technology has been widely used in biological significance interpretation, food safety and quality, breeding, disease diagnosis, functional compound identification, and other fields. Its application in poultry science has also become the focus of widespread attention. With the sustainable development of analytical techniques, metabolomics has great potential in the application of poultry science. In this paper, the research progress of metabolomics in poultry growth and development, genetics and breeding, egg quality, meat quality, and disease is reviewed and concluded, which is expected to provide scientific ideas for the research of metabolomics in poultry.

## 1 Introduction

Poultry meat and eggs are welcomed by consumers because of their rich nutritional value, low price, low fat, and cholesterol content (1). Therefore, with an increasing demand for poultry products, there is more and more research on the production technologies of healthy and high-quality poultry products.

**Figure 1:**
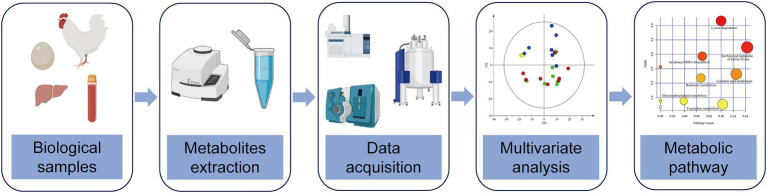
Schematic diagram of the technical route of poultry metabolomics, including biological samples selection, metabolites extraction, data acquisition, multivariate analysis and metabolic pathway.

Metabolomics is a branch of omics developed in the mid-1990s, and the concept comes from the metabolome. Metabolomics refers to qualitatively and quantitatively analyzing small molecular metabolites (≤1,000 Da) in cells, tissue, organ, or organism, which reflect the metabolic pathway of endogenous metabolites affected by internal and external factors (2). With the characteristics of high throughput and high sensitivity, metabolomics is developed after genomics and proteomics and is called the peak of the trilogy of genomics (3). According to the analysis scope, metabolomics can be divided into two types: non-targeted metabolomics and targeted metabolomics. Non-targeted metabolomics aims at all endogenous metabolites in the organism, while targeted metabolomics aims at several target compounds (4, 5). Targeted metabolomics has better sensitivity and specificity than non-targeted metabolomics, so targeted metabolomics can verify and expand the results of non-targeted metabolomics analysis. At present, the commonly used analytical techniques include nuclear magnetic resonance (NMR), gas chromatography coupled to mass spectrometry (GC-MS), and liquid chromatography coupled with mass spectrometry (LC-MS). The comparison between the three analytical techniques showed that the sensitivity of NMR is lower than that of LC-MS, but its repeatability is very high. GC-MS has preferably been applied to analyze volatile metabolites such as fatty acids with sample derivatization pretreatment. With high sensitivity, high throughput and wide range, LC-MS analytical platform is becoming increasingly popular in the field of metabolomics. Researchers usually choose different analytical techniques according to the experimental needs. The basic technical route of poultry metabolomics is shown below (Figure 1).

Metabolomics is widely used in various fields, such as disease diagnosis and mechanism (6–8), drug development (9, 10), nutrition science (11, 12), microbial research (13, 14) and food science. With the continuous development of metabolomics, it has been well applied in physiology, pathology, nutrition and food safety in poultry science. Metabolomics analysis of poultry serum, muscle, kidney, liver, and egg products can reflect health status, meat quality, egg quality, pathological mechanism, and so on (Table 1). This will be beneficial to the work of farmers or the researchers, and provide a strong guarantee for health. Xiao et al. performed the ^1^H-NMR method to examine the metabolic composition of Wuding chicken of different ages, so as to evaluate the quality of chicken meat (15). In order to quickly and sensitively identify whether the meat product was mixed with other kinds of meat, some researchers used high-resolution MS for experimental analysis (16). In summary, the application of metabolomics technology in poultry has great potential.

**Table 1:** Application of different metabolomics platforms to different samples from different poultry species.

Species	Sample	Platform	Metabolites No.	Main metabolites or classification	Pathways	References
Duck	Breast muscle	LC-MS	499	Carnosine, inosinic acid, 5-hydroxy indole acetic acid, 1-O-sinapoyl-beta-d-glucose	Aminoacyl alanine, aspartate and glutamate metabolism, purine metabolism	Liu et al. (66)
	Liver	UHPLC-MS	91	Lipids and lipid-like molecules, organic oxygen compounds, organic acids and derivatives, organoheterocyclic compounds	Biosynthesis of unsaturated fatty acids, linoleic acid metabolism, ATP-binding cassette (ABC) transporters	Zhang et al. (67)
	Egg yolk	UHPLC-MS/MS GC-TOF-MS	1,205	Monoolein, emodin, flavonoids, imethylethanolamine, harmalan, amino acids and dipeptides	Lipid metabolism	Tian et al. (68)
	Egg	UHPLC-HRMS	14	N-behenoyl-d-erythro-sphingosine, 1,2-dipalmitoyl-sn-glycero-3-phosphocholine, n-nervonoyl-d-erythro-sphingosine	/	Dong et al. (34)
Goose	Liver, intestinal contents	GC-TOF-MS	324, 314	Fatty acids, amino acids, organic acids, amines	Glycolysis/gluconeogenesis, glycerolipid metabolism, fatty acid degradation, biosynthesis of unsaturated fatty acids	Zhao et al. (69)
	Follicle	LC-MS/MS	333	Amino acids, lipids, fatty acids, nucleotides, organic acids	Steroid biosynthesis, sulfur metabolism, galactose metabolism, ABC transporters, Pantothenate and CoA biosynthesis	Yuan et al. (70)
	Serum	GC-TOF/MS	185	Pyruvic acid, alanine, proline, beta-glycerophosphoric acid, lactic acid	Amino acids metabolism, carbohydrate metabolism, lipid metabolism	Gong et al. (71)
Quail	Serum, stool	LC-MS/MS	9, 94	Stearic acid, nonanedioic acid, 1-acyl-sn-glycerol 3-phosphate, 1-acylglycero-phosphoinositol, l-palmitoylcarnitine,	Glycerophospholipid metabolism	Bian et al. (72)
	Serum	GC-MS	165	Beta-alanine, L-serine, acetoacetic acid, succinic acid, 3-aminoisobutanoic acid, urea, 1-butylamine	Ketone body metabolism, butyrate metabolism, arginine and proline metabolism	Pu et al. (73)
Dove	Plasma, pectoralis, liver, adipose, kidney, gastrocnemius	LC-MS	154, 123, 125, 92, 188, 105	Acylamide, myoinositol, norvaline, 4-aminobutyric acid, 2-deoxyadenosine, 2-hydroxybenzoic acid	Pantothenate and coa biosynthesis, inositol phosphate metabolism, valine, leucine and isoleucine biosynthesis, butanoate metabolism	Mohr et al. (74)
Chicken	Cecal contents	UHPLC-MS/MS	1,744	Glutamic acid, pantothenate acid, N-acetyl-L-aspartic acid	Amino acid metabolism lipid metabolism	Zhang et al. (75)
	Egg	^1^H NMR	/	Triacylglycerols, phospholipids, cholesterol	/	Cardoso et al. (76)
	Breast meat	2D qNMR	114	Asparagine, inosinic acid, anserine, lacyic acid, alpha-d-glucose, nicotinic acid	Purine metabolism, primary bile acid biosynthesis, arginine and proline metabolism	Kim et al. (77)
	Breast	^1^H-NMR	56	Lactate, creatine, carnosine	Cysteine and methionine metabolism, nicotinate and nicotinamide metabolism, aurine and hypotaurine metabolism, glycerophospholipid metabolism	Consolo et al. (48)
	Chilled breast	UHPLC-MS/MS	265	Indole-3-carboxaldehyde, uridine monophosphate, s-phenylmercapturic acid, gluconic acid, tyramine, serylphenylalanine	Glycine and serine metabolism, betaine metabolism, glucose-alanine cycle, phosphatidylcholine biosynthesis	Zhang et al. (78)
	Fresh meat	UPLC-QQQ-MS	10	Acetylcholine, Methionine, Proline, Valine, Norleucine	Valine, leucine and isoleucine biosynthesis, glycerophospholipid metabolism, histidine metabolism	Chen et al. (79)

LC-MS, liquid chromatography mass spectrometry; UHPLC-MS/MS, ultra-high-performance liquid chromatography-mass spectrometry; GC-TOF/MS, gas chromatography time-of-flight/mass spectrometry; UHPLC-HRMS, ultra high performance liquid chromatography coupled with high resolution mass spectrometry; LC-MS/MS, liquid chromatography tandem mass spectrometry; GC-MS, gas chromatography mass spectrometry; ^1^H NMR, Nuclear magnetic resonance of hydrogen; 2D qNMR, Two-dimensional quantitative NMR spectroscopy; UPLC-QQQ-MS, ultra-performance liquid chromatography coupled with triple-quadrupole tandem mass spectrometry.

The following chapters will mainly introduce the application of metabolomics in poultry growth and development, genetics and breeding, disease prevention and control, egg quality, and meat quality. This review is focused on the cutting-edge research of metabolomics in the field of poultry science in recent years, which will help to provide a reference for the further development of poultry science.

## 2 Metabolomics in the growth and development of poultry

**Figure 2:**
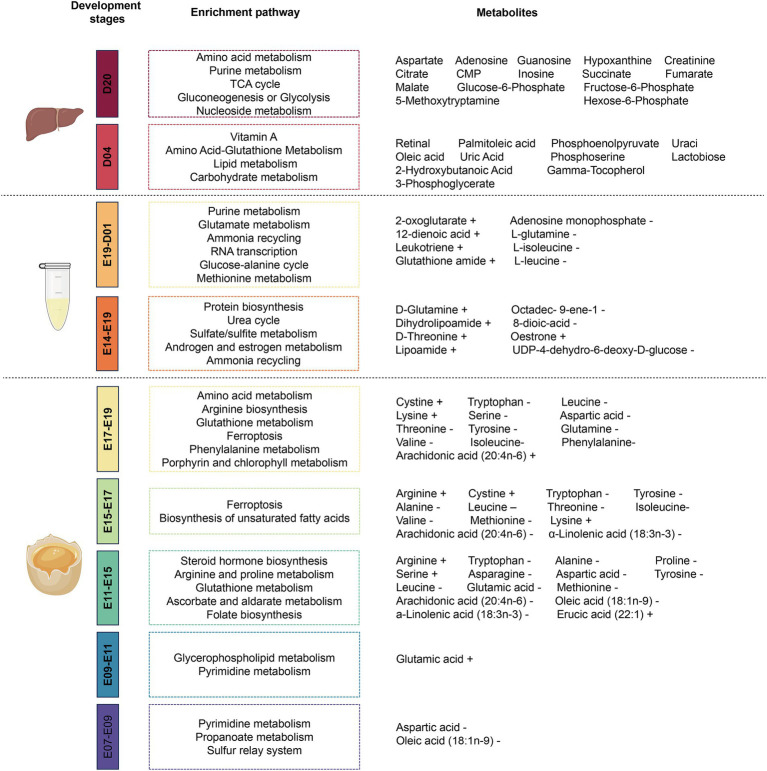
Metabolites changing at different developmental stages in poultry. The changes of egg yolk in the five stages of E07-E09, E09-E11, E11-E15, E15-E17, and E17-E19, serum in the two stages of E14-E19 and E19-D01, and liver metabolites and metabolic pathways in the two stages of D04 and D20. E07 indicates the seventh day of unhatched and D01 indicates the first day after hatching.

Through the detection of metabolites in serum, egg yolk, liver, and other tissues, some small molecular metabolites can be screened and selected as biomarkers for different breeds and developmental stages of poultry. It not only provides a theoretical basis for the different phenotypic traits of poultry across different breeds and at different growth stages, but also offers new insights into the changes in metabolites at these growth stages. The changes of tissue metabolites and metabolic pathways at different growth stages are shown in Figure 2.

The hatching egg is a complex structure, and the growing breeding chicken embryo spends more than 30% of its life cycle in it. As the first diet of the early embryo, the nutrients in the hatching egg play a crucial role in the growth and development of chicks and the improvement of immune function (17). The yolk part of the hatching egg structure is considered to be the main nutrient provider throughout the development of the embryo.

Liu et al. used UHPLC-MS/MS metabolomics to study the changes of yolk metabolites on the 7th, 9th, 11th, 15th, 17th, and 19th day of the embryo. It was found that the yolk metabolites were changed when compared with other days of the embryo, and most of the metabolites were annotated in the amino acid metabolic pathway on the 11th, 15th, 17th, and 19th day of the embryo (18). It can be seen from the above results that the dynamic monitoring of metabolites (nutrient providers) in egg yolk can help understand the nutritional requirements of embryos at different developmental stages. Therefore, metabolomics technology can provide precise nutrition with benefits to promote healthier and stronger chicks during hatching.

It is worth noting that the lipid nutrients in egg yolk are mainly stored in the liver in the form of cholesterol esters after being ingested and absorbed by the embryo (19). The accumulation of lipids accounts for 10% of the mass of the liver until 3 days before breaking the shell (20). After hatching, the chicks use the remaining egg yolk and stored lipids as nutrients for their growth. However, after absorption and consumption on the fifth day after birth, the fat mass in the liver was only one-tenth of the initial storage (21). And the metabolic burden on the liver and how to quickly and effectively maintain a stable energy balance are the main problems faced by the chicks after birth. With the application of high-throughput metabolomics and combined with transcriptome, a study was performed to monitor the liver metabolic network under natural conditions on day 4 (D4) and day 20 (D20) after hatching (22). It was observed that the liver of D4 had a feature of hypoxia, and the TCA cycle was inhibited as compared with that of D20. These results might be due to the mismatch between the rapid growth of liver cells and the delayed formation of blood vessels. Meanwhile, in the liver on D4 from the chicken, pyruvate metabolism and 3-phosphoglycerate metabolism became important metabolic forks under hypoxia conditions, and the polyamine synthesis pathway was enriched and promoted the increase of urea level. During the anaerobic phase of D20, the metabolic pattern in the liver shifted from protecting on its own growth mode to supporting metabolic mode in other tissues, and the enrichment of proline and lysine metabolites suggested the increase of collagen generation and crosslinking in the liver at this stage. At the same time, the anaerobic effect of carbohydrate metabolism was reduced, glucose storage and fatty acid synthesis were increased, and the liver was gradually transformed into a mature liver. The comparison between the two time points from the metabolomic results provided insights into the regulation of metabolites in the liver during the transition from an anoxic to oxic environment in the chicken.

Peng et al. used LC/MS-QTOF based metabolomics to determine the changes of main metabolites in the serum on the 14th and 19th day of embryo incubation and 1 day after hatching, and analyze the related pathways of the differential metabolites during chicken embryo development (23). It was found that 39 metabolites changed from the 14th to 19th day of embryo incubation, and 68 metabolites changed significantly from the 19th day of embryo incubation to the first day after hatching. Among them, the changes in serum glutamine, threonine, and oestrone contents could be used as candidate indicators for the evaluation of early embryonic development because they contributed to promoting the growth and development of chicken embryos from 14 to 19 days old. Additionally, the metabolomic results revealed that L-glutamine, L-isoleucine, and L-leucine could be used as early feed additives for broilers to meet their early growth needs. The above metabolomic results provided insights into the role of serum metabolites in different stages of chicken embryo development. Therefore, metabolomics can be used to screen differential metabolites in the process of growth and development in poultry, which can be treated as biomarkers, indicators or feed additives to explain the mechanism of growth and development and meet the needs of their growth and development.

In the above study, both UHPLC-MS/MS and GC-TOF MS technologies were employed. UHPLC-MS/MS is suitable for detecting a wide range of metabolites, including amino acids and fatty acids, offering highly accurate quantitative analysis. GC-TOF MS is particularly suited for primary metabolites like sugars and short-chain fatty acids, making it ideal for high-throughput analysis. Moreover, Peng et al. employed quality control samples to enhance the reliability of data analysis, thus reducing data fluctuations caused by experimental errors, which is essential for the analysis of high-throughput samples. Therefore, selecting the appropriate technology based on the specific type of target metabolites and experimental requirements is vital for studying the changes in metabolomics during poultry growth and development.

## 3 Metabolomics in poultry product quality

Eggs are highly nutritious, containing essential amino acids, macroelements, microelements, vitamins, etc., and are considered indispensable food for humans (1, 24). Metabolomics technology offers a powerful means of identifying differential metabolites related to egg quality, thus providing insights into enhancing egg quality.

Previous studies have shown that the yolk is rich in free fatty acids and different types of lipids (25, 26). The lipids can significantly enrich the neuronal and subcellular structure of the hippocampus, which plays an important role in brain development and cognition (27). But the extreme claims about dietary yolks increasing cholesterol risk have gradually overshadowed the physiological value of lipids (28, 29). In order to change this bias, Paul et al. used high-resolution MS to characterize the complex structure of lipids in order to clarify the potential nutrients of lipids in egg yolk (30). The results showed that egg yolk is rich in phospholipids, ceramides and triglycerides, and structural lipids have *ω*-3 and ω-6 fatty acids, which play an important role as essential precursors of endogenous anti-inflammatory lipid mediators. Interestingly, the yolk structure glycerophospholipids could reduce the absorption of dietary cholesterol in the view of cholesterol risk (31–33). In fact, the nutrient composition in the yolk changes dynamically with the continuous metabolism of the yolk sac, which becomes an important factor affecting the embryo development of broilers. Dong et al. employed a non-targeted metabolomics approach utilizing ultra high-performance liquid chromatography coupled with high resolution mass spectrometry (UHPLC-HRMS) to profile the metabolites of sea duck and caged duck eggs (34). It was observed that the levels of n-behenoyl-d-erythro-sphingosine, 1,2-dipalmitoyl-sn-glycero-3-phosphocholine, and n-nervonoyl-d-erythro-sphingosine in sea duck eggs were significantly higher than those in the caged duck eggs. These findings provided robust evidence for the identification of sea duck eggs and effective means to combat fraud in the poultry and egg industry. Goto et al. utilized metabolomics technology to explore the effects of hen breed and feed conditions on egg composition (35). The researchers analyzed 138 yolk metabolites and 132 albumen metabolites using the GC-MS method. The study revealed that feed conditions significantly altered the levels of three yolk metabolites (erythritol, threitol, and urea) and 12 albumen metabolites (erythritol, threitol, ribitol, linoleic acid, isoleucine, dihydrouracil, 4-hydroxyphenyllactic acid, alanine, glycine, N-butyrylglycine, pyruvic acid, and valine). These findings indicate that genetic and environmental factors play a vital role in the composition of eggs, thereby offering a basis for designing eggs that meet the nutritional needs of consumers in the future. Liu et al. employed LC-MS based metabolomics to analyze the albumen and yolk of long-term stored duck eggs and fresh duck eggs (36). For duck eggs, the long-term storage resulted in the degradation of nutrients such as amino acids, fatty acids, nucleotides, sugars, and vitamins, and the production of harmful substances such as ammonia and biogenic amines, which ultimately compromised the quality of duck eggs. So the consumption of long-term stored duck eggs will have a negative impact on health. In summary, at the level of metabolites, metabolomics technology provides evidence and insights into enhancing the nutritional value, quality and safety of eggs.

Eggshell quality is a crucial factor in poultry egg production, with eggshell rupture accounting for a significant loss of total egg yield, typically around 8–10% (37). The eggshell, which takes approximately 19 h to form, is a highly organized and well-calcified structure that consists of large quantities of calcium, carbonate, proteins, and other chemicals released by the expanding uterus as the egg passes through the isthmus (37–39). Deposited in large quantities and highly organized, these substances constitute the main components of the eggshell. Endogenous metabolites present in the uterine fluid play a vital role in the deposition of calcium carbonate and the subsequent calcification of the eggshell. To investigate whether metabolite differences in the intra-uterine fluid significantly impact eggshell quality, Wang et al. utilized the LC-MS metabolomics approach to identify the metabolites in the uterine fluid of chickens that produced eggs with varying eggshell quality (40). The results revealed that phosphatidylcholine, diacylglycerol, verapamil, risedronate, coproporphyrinogen III, biliverdin phosphatidylcholine, diacylglycerol, verapamil, and biliverdin are essential components involved in eggshell calcification. In addition, it was found that eutectic protein 3 and biliverdin could enhance eggshell strength. Regulating the composition of uterine fluid could potentially improve eggshell quality, and the metabolomics analysis opens up new avenues to increase economic returns in poultry farms.

As a kind of high-quality protein, poultry meat is easy to be absorbed and used by the human body. In recent years, the quality of poultry meat is a research focus. Metabolomics technology can deeply understand the correlation between meat quality and breed, day age and storage time, and then screen and label differential metabolites related to meat quality, which is helpful to improve the speed and accuracy of key metabolite identification and establish a high-quality evaluation system for meat quality.

Shi et al. used LC-MS/MS metabolomics to explore the effect of selective breeding on chicken quality (41). The results showed that glycerol phospholipid metabolism played an important role in chicken flavor and development and affected chicken quality. Li et al. detected the dynamic changes of metabolites in chicken breast meat during different growth and development stages by LC-MS/MS metabolomics (42). In this study, 573 metabolites were identified in five age stages, which directly reflected the nutritional changes of chicken meat and helped to understand the biological process in the development of meat quality. With the application of NMR-based metabolomics, Xiao et al. evaluated the chemical components in the muscle of Wuding chickens at the age of 110, 140, 170, 200, and 230d (15). The total metabolite content in the chicken breast and leg meat was significantly higher in the other four periods compared to 110 days. Moreover, the levels of lactic acid, creatine, inosinic acid, glucose, and other precursors were different in the chest muscle and leg muscle at 230d. The findings were significant to further understand the relationship between chicken metabolism and age, and also could be used to evaluate chicken meat quality.

The flavor and nutritional value of the poultry in the process of storage is also one of the focused issues. Frozen broilers may rot before sale due to the rampant growth of microorganisms and the erosion of spoilage bacteria. The rotten frozen chicken will devour a large amount of water and nutrients and produce excess harmful metabolites. In order to characterize the microbial species and metabolite profile of frozen chicken, Zhang et al. used 16S rRNA gene sequencing and UHPLC-MS/MS non-targeted metabonomic techniques to monitor and observe frozen chicken samples (43). The results showed that *Acinetobacter*, *Serratia*, *Curella*, *Shivella*, and *Obesobacter* were the main bacteria causing spoilage of frozen chicken, and 10 metabolic pathways such as histidine metabolism and purine metabolism were the potential pathways to lead to spoilage of frozen chicken. This study was conducive to understanding the changes of metabolites in frozen chickens during storage and provided a basis for the development of new methods for detecting the freshness of frozen chickens. In addition to the traditional metabonomic techniques, Zhou et al. developed a new metabolomic method with the employment of laser ablation electrospray ionization mass spectrometry (LAESI-MS), for direct analysis and identification of meat samples without sample pretreatment (44). Combined with the use of principal component analysis and partial least squares discriminant analysis, the metabolomics technique based on LAESI-MS was proved to be a rapid and accurate method for the distinction of meat samples including chicken, duck, pork, beef, and mutton.

Metabolomics, with its high sensitivity, high throughput, and robustness, has emerged as a powerful tool for both the identification and quality assessment of poultry products such as poultry meat and egg. Screening and labeling of differential metabolites related to meat and egg quality requires statistical analysis of metabolomics data. Multivariate analysis (PCA, PLS-DA, or OPLS-DA) was used in all of the above studies to identify significant metabolites and to analyze between-group differences, and VIP values and *p*-values from *t*-tests were used to screen for significant metabolites. The focus on data handling varies from study to study. For instance, Goto et al. analyzed the data by using a two-way mixed-design analysis of variance (ANOVA), and the raw data in the other two articles were transformed by ProteoWizard and the XCMS project was employed for peak alignment, retention time correction as well as peak area extraction. With continuous advances in metabolomics technology, it is crucial to explore simpler and more effective data processing methods to investigate the molecular mechanisms behind egg quality and poultry meat characteristics.

## 4 Metabolomics in poultry breeding

Poultry with excellent breeds and strong production performance is welcomed by more farms. Genetics and breeding are of great significance in increasing production level and efficiency. In order to improve the selection efficiency and speed up the genetic process, molecular genetic markers can be used to assist poultry breed selection.

Liu et al. used LC-MS to analyze the metabolites of Chahua Chicken No. 2 (CH) and Yao Chicken (Y) (45). The results showed that CH and Y had 85 different metabolites involved in the oxidation of amino acids and fatty acids, which could provide the basis for the molecular breeding of chicken quality traits in the future. Ji et al. used LC-MS based metabolomics to detect the metabolite profile in the adipose tissue of lean Leghorn and Fayoumi chickens, and compared the results to the commercial broiler chickens to explore the pathways associated with heritable leanness in chickens (46). Metabolomic results found that the lipolysis and fatty acid oxidation in white adipose were up-regulated in the lean chickens, indicating the essential roles of these pathways in heritable differences in fatness.

Both studies utilized metabolomics and gene expression profiling, but focused on different aspects. Liu et al. provided a scientific foundation for genetic improvement and breeding of local chicken breeds, emphasizing the potential of the identified genes for improving meat quality and fat deposition in poultry. Ji et al. offered insight into the mechanisms underlying leanness in chickens, suggesting potential pathways for future research on obesity and adipose metabolism in humans. The study also highlights chickens as a unique model for studying human obesity and metabolic disorders.

## 5 Metabolomics in poultry disease

**Figure 3:**
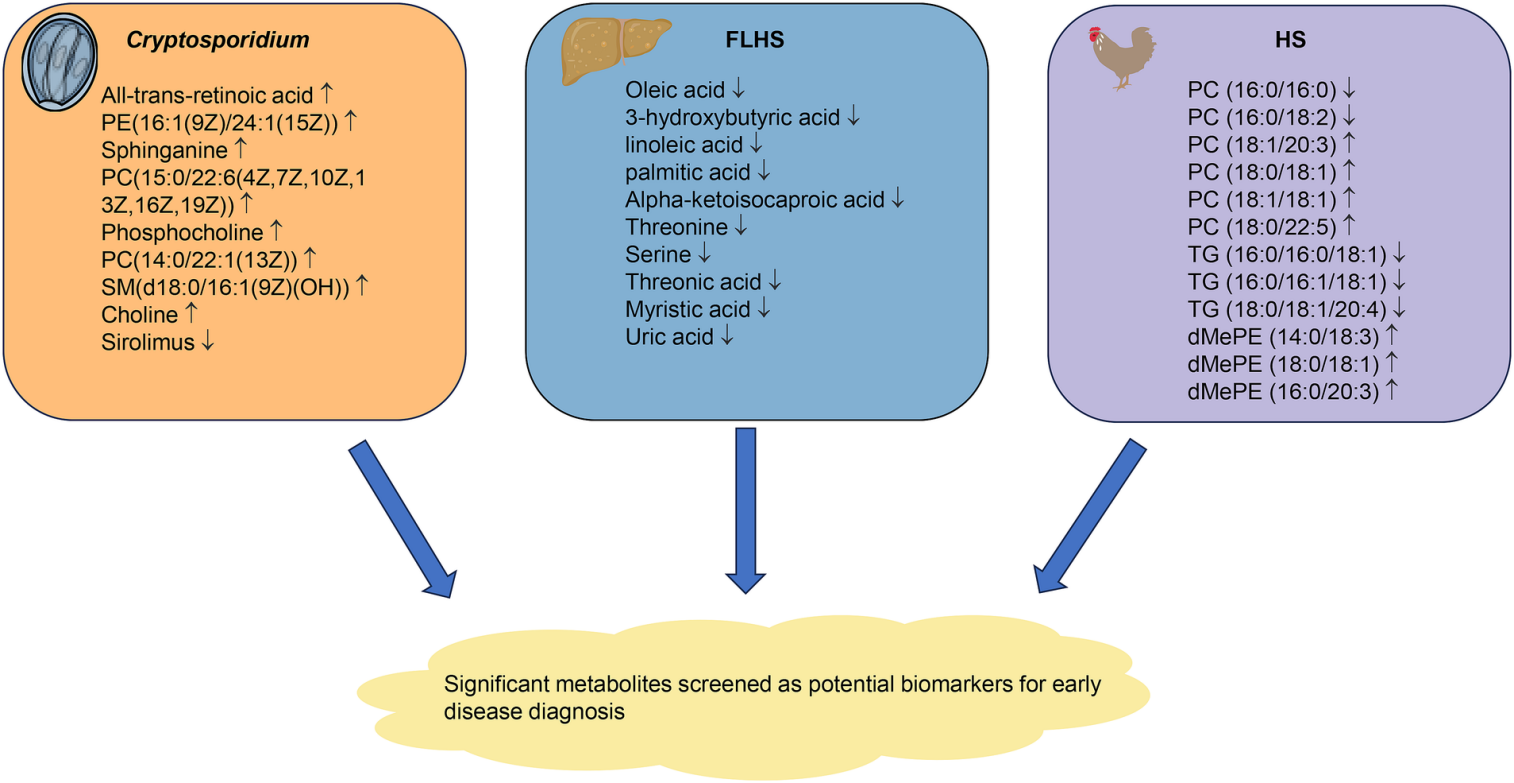
Metabolomics technology screens for significantly different metabolites that can be used as biomarkers for early diagnosis of disease. The changes of metabolites of cryptosporidiosis, FLHS and HS are shown in the figure. FLHS, fatty liver hemorrhage syndrome; HS, heat stress.

Poultry disease is the most disturbing thing in the process of poultry breeding. Once the disease breaks out, it will affect a large number of birds and bring huge economic losses to the farmers. There are still some problems such as difficulty and high cost in the prevention and treatment of poultry diseases. Metabolomics has been proven to have great potential in predicting the incidence, severity, and progression of the disease (47–49). With the development of an analysis platform, metabolomics technology can be used to screen differential metabolites as markers for the early detection and evaluation of diseases (Figure 3).

As one of the most harmful zoonotic parasitic diseases, *Cryptosporidium* was first discovered in the early 20th century. It can cause gastroenteritis symptoms in a variety of vertebrates. In poultry and birds, some *Cryptosporidium* subtypes induce obvious respiratory symptoms such as cough, runny nose and dyspnea, and even cause death in severe cases (50). In addition, the study found that *Cryptosporidium* infection can lead to the suppression of the host immune system, resulting in increased susceptibility to avian influenza, Newcastle disease, and other diseases (51). Metabolomics have been used to detect the iconic metabolites in infected chickens. Wu et al. used UPLC-MS metabolomic technique and multivariate statistical analysis to study the metabolic characteristics of serum samples of chickens infected with *Cryptosporidium baileyi* in order to distinguish the potential metabolites between infected and uninfected chickens (52). The results showed metabolomics profiles in the serum were significantly changed after the infection of *Cryptosporidium baileyi,* and a total of 138 differential serum metabolites enriched in energy, lipid metabolism, and immune-related pathways were detected. Notably, it was found that nine metabolites such as choline, all-trans retinoic acid, phosphocholine and sphinganine could be used as serum diagnostic markers after *Cryptosporidium baileyi* infection, which provided the information for exploring the invasion mechanism of *Cryptosporidium* and the pathological changes of the host.

Fatty liver hemorrhage syndrome (FLHS) is a nutritional metabolic disease characterized by lipid metabolism disorders. Its clinical manifestations are fat deposition, rupture, and bleeding in the liver (53, 54). Mostly occurring in caged laying hens, FLHS leads to a decrease in egg production, the shortening of the peak period of egg production, and the increase in mortality, which causes huge economic losses to the layer industry (55). Guo et al. found that metabolomics technology could be applied to the clinical diagnosis of FLHS in laying hens, which provided the potential biomarkers for further understanding the development of FLHS (56). Serum metabolites of the laying hens with FLHS and the healthy control were analyzed by GC-TOF-MS at two-time points (40 and 80 days). Forty different metabolites were screened, which were mainly related to lipid metabolism, amino acid metabolism, and energy metabolism disorders. These studies proved that metabolomics is a powerful tool to study and identify the diagnostic biomarkers related to FLHS in laying hens, and provide new insights into its biological mechanism. Meng et al. revealed the potential pathogenesis of FLHS and abnormal arachidonic acid metabolism in laying hens by applying non-targeted and targeted metabolomics based on UPLC-QTOF/MS (57). Through the analysis of the changes of the liver metabolic profile, 42 liver metabolites were found. Pathway analysis of these liver metabolites revealed that the disorder of arachidonic acid metabolism had an important role in the pathogenesis of FLHS.

For the growing environment, temperature plays an important role in the growth and metabolism of poultry. Heat stress (HS) is an important factor affecting poultry production in tropical and subtropical areas, which mainly causes the decrease in feed intake and growth performance of broilers (58), and leads to pathological changes such as abnormal lipid metabolism and secretion disorder of ketone body components (59, 60). HS can cause fat deposition in broilers, inhibit the proliferation of poultry liver cells, and induce liver cell apoptosis. In order to clarify the metabolomic-level mechanism of the influence of HS, Guo et al. used LC-MS based metabolomics to examine the liver lipid metabolites of Chinese indigenous slow-growing broilers (Huaixiang chickens) (61). It was found that HS could change the content levels of 12 hepatic lipid metabolites mainly including phosphatidylcholine, triglyceride and dimethyl-phosphatidyl ethanolamine. Meanwhile, pathway analysis found that HS altered the pathways of linoleic acid, alpha-linolenic acid, glycerolipid and glycerophospholipid metabolism in the liver of broiler chickens.

It is worth noting that corticosterone (CORT) is one of the stress hormones in multiple types of poultry stress including HS (62). Stressors can trigger the hypothalamic-pituitary-adrenal axis and produce large amounts of CORT in chickens (62, 63). Excessive production of CORT can damage the immune system of chickens and negatively affect the growth and production performance (64). In order to explore the effects of physiological stress brought by CORT on metabolic groups of the liver, kidney, and breast muscle of chickens, a chicken stress model was established by administering CORT (dissolved in ethanol) in drinking water (65). With the application of an untargeted ^1^H-NMR-based metabolomics approach, it was found that CORT, respectively, altered 11, 46, and 14 metabolites in the liver, kidney and breast muscle, which were linked to amino acid and sugar metabolism. In addition, some metabolites such as dimethylglycine, galactose, and carnosine in breast muscle were regulated by CORT, which might be associated with meat quality. In summary, the identification of metabolites as biomarkers of stress will help to develop and evaluate mitigation strategies to enhance bird health.

The studies mentioned above suggest that metabolomics can identify metabolic differences between diseased and healthy animals. The selection of samples for testing depends on the specific disease being investigated. For example, serum samples are used for diagnosing Cryptosporidium infection, serum and liver metabolites are analyzed for FLHS, and liver lipid metabolites are tested for HS. Disease-specific biomarkers vary, and the metabolome can be influenced by factors such as physiological states, environmental stimuli, and other variables. Thus, when using metabolomics for early disease diagnosis and prevention in poultry, it is critical to select samples that accurately reflect the current physiological state.

## 6 Conclusion

As an emerging technology, metabolomics is employed to identify and monitor the changes of small-molecule metabolites in organisms caused by exogenous disturbances, which will help to explore the metabolic mechanism. The wide applications of metabolomics technology in poultry disease diagnosis, genetics and breeding, growth and development, egg quality, and meat quality detection indicate this approach is highly promising. However, there are still many problems to be solved in the current metabolomics technology, for example, there are still some limitations in the sensitivity and coverage of some detection instruments, and the metabolites detected may only be a small part of the gene variation. Therefore, the sensitivity, stability, specificity, and broad spectrum of the detection technology still need to be further improved. Moreover, multi-omics technology is the future trend that the integration of genomics, transcriptomics, proteomics and metabolomics can provide comprehensive information at the different levels for poultry research.

As omics technologies continue to evolve, metabolomics is increasingly recognized as a crucial tool in poultry research. The future development of this field will likely be propelled by advancements in metabolite detection instruments, improvements in metabolite databases, and the widespread application of diverse metabolomics analysis strategies. To address the challenges posed by high-dimensional, complex datasets in current metabolomics analyses, it will be essential to establish a more comprehensive and standardized reference database, enabling the effective use of data from many unknown metabolites. Furthermore, future research trends are expected to focus on integrating multiple analytical platforms to enable a more holistic analysis of metabolomics data from various dimensions, offering deeper insights into poultry metabolic mechanisms. At the same time, the rapid development of artificial intelligence technologies will significantly enhance the ability to process metabolomics data, providing innovative approaches for dimensionality reduction and more nuanced interpretations of metabolomics information.
